# Focus on the Complex Interconnection between Cancer, Narcolepsy and Other Neurodegenerative Diseases: A Possible Case of Orexin-Dependent Inverse Comorbidity

**DOI:** 10.3390/cancers13112612

**Published:** 2021-05-26

**Authors:** Maria P. Mogavero, Alessandro Silvani, Lourdes M. DelRosso, Michele Salemi, Raffaele Ferri

**Affiliations:** 1 Istituti Clinici Scientifici Maugeri, IRCCS, Scientific Institute of Pavia, 27100 Pavia, Italy; paola_mogavero@libero.it; 2 Department of Biomedical and Neuromotor Sciences (DIBINEM), University of Bologna, 40126 Bologna, Italy; alessandro.silvani3@unibo.it; 3 Seattle Children’s Hospital and University of Washington, Seattle, WA 98105, USA; Lourdes.DelRosso@seattlechildrens.org; 4 Oasi Research Institute—IRCCS, 94018 Troina, Italy; msalemi@oasi.en.it

**Keywords:** cancer, neurodegeneration, orexin, narcolepsy, inverse comorbidity

## Abstract

**Simple Summary:**

This narrative review first describes from several points of view the complex interrelationship between cancer and neurodegeneration, with special attention to the mechanisms that might underlie an inverse relationship between them. In particular, the mechanisms that might induce an imbalance between cell apoptotic and proliferative stimuli are discussed. Second, the review summarizes findings on orexins and their involvement in narcolepsy, neurodegenerative diseases, and cancer, starting from epidemiological data then addressing laboratory findings, animal models, and human clinical observational and interventional investigations. Important research efforts are warranted on these topics, as they might lead to novel therapeutic approaches to both neurodegenerative diseases and cancer.

**Abstract:**

Conditions such as Alzheimer’s (AD) and Parkinson’s diseases (PD) are less prevalent in cancer survivors and, overall, cancer is less prevalent in subjects with these neurodegenerative disorders. This seems to suggest that a propensity towards one type of disease may decrease the risk of the other. In addition to epidemiologic data, there is also evidence of a complex biological interconnection, with genes, proteins, and pathways often showing opposite dysregulation in cancer and neurodegenerative diseases. In this narrative review, we focus on the possible role played by orexin signaling, which is altered in patients with narcolepsy type 1 and in those with AD and PD, and which has been linked to β-amyloid brain levels and inflammation in mouse models and to cancer in cell lines. Taken together, these lines of evidence depict a possible case of inverse comorbidity between cancer and neurodegenerative disorders, with a role played by orexins. These considerations suggest a therapeutic potential of orexin modulation in diverse pathologies such as narcolepsy, neurodegenerative disorders, and cancer.

## 1. Cancer and Neurodegenerative Diseases

The World Health Organization (WHO) highlights cancer as one of the most common causes of death, which accounted for almost 10 million deaths worldwide in 2020 [1]. Overall, estimates indicate that one in five persons will get cancer in their lifetime before 75 years of age and one in ten will die from the disease.

The incidence and prevalence of neurodegenerative diseases are also high. Alzheimer’s disease (AD) is a neurodegenerative disorder characterized by brain β-amyloid plaques and neurofibrillary tangles formed by phosphorylated tau (P-Tau) protein deposits. AD is the most frequent neurodegenerative disease and affects 24 million people worldwide. The second most frequent neurodegenerative disease is Parkinson’s disease (PD), characterized by Lewy bodies and neurites formed by alpha-synuclein deposits. PD has a prevalence of 1% in people older than age 60, and of 3% in people aged 80 years or older [2]. There is also increasing interest, in the literature, on multiple sclerosis (MS). Despite being an inflammatory demyelinating disease, MS can also be viewed as a neurodegenerative disease because of the cascade of events triggered by neuroinflammation and leading to neurodegeneration [3,4,5]. The key elements that induce neurodegeneration include activation of microglia, chronic oxidative damage, and altered mitochondrial function in axons, leading to chronic cellular stress and imbalance of ion homeostasis, resulting in axonal and neuronal death [6]. The total prevalence of MS in Europe is 83 per 100,000, which is lower than the prevalence of AD and PD, but still associated with substantial social and economic costs [7]. Amyotrophic lateral sclerosis (ALS), which is characterized by the progressive loss of motor neurons in the brain and spinal cord, is also rare, with a prevalence of 2–3 per 100,000 [8].

### 1.1. The Connection between Cancer and Neurodegenerative Diseases

The incidence of many common cancers and neurodegenerative diseases including AD and PD increases with age [9,10]. MS also typically occurs in adults, although its prevalence peaks for subjects between 35 and 64 years of age and does not further increase thereafter [7]. Similarly, the risk for ALS peaks at 50–75 years of age [8]. It is essential to focus attention on the development of alternative treatments that address age-related diseases, also in consideration of the increase in age in the population.

Despite the common age-related trends in the incidence of cancer and neurodegenerative disorders, a meta-analysis of observational studies including 577,013 participants concluded that there was a significantly lower co-occurrence of cancer in patients with neurodegenerative disorders. In particular, patients with AD had a markedly reduced co-occurrence of cancer in general, but no data were available for specific cancers [11]. The Framingham heart study, a longitudinal community based cohort study, in which 221 cases were evaluated for a 10-year follow-up, also indicated a lower risk of AD for cancer survivors: the risk of AD was lower in survivors of smoking-related cancers; this model for cancer is similar to that seen in PD and suggests an inverse association between cancer and neurodegeneration; the effect was stronger for lung cancer and preserved for participants who survived at least to 80 years of age [12]. Furthermore, in evaluating the interconnection between AD and lung cancer, it is important to consider that cigarette smoking appears to play a neuroprotective influence for both AD and PD [13], while it represents a known risk factor for cancer of the lung.

A recent systematic review and meta-analysis confirmed a weak but significant decrease in AD risk comparing older adults with vs. without a previous cancer diagnosis, but it could not rule out a role of survival bias [14]. Epidemiological studies indicate that AD patients have a lower risk of developing lung cancer and a higher risk of developing glioblastoma: transcriptomic meta-analyses reveal a significant number of genes with reverse expression patterns in AD and lung cancer, compared to AD and glioblastoma [15]. Meta-analytical data indicate that patients with PD and MS have a reduced co-occurrence of lung and prostate cancers, and patients with PD also have reduced co-occurrence of colorectal cancer. These associations are not consistent across all cancer types, with an increased cooccurrence of melanoma in patients with PD and of brain cancers in patients with MS [11]. On the other hand, a recent population-based case-control study reported negative point estimates of the odds of developing PD in survivors of most cancers, with the notable exception of skin and female breast cancer but with wide confidence intervals overlapping with zero [16]. A recent meta-analysis has shown that patients with MS have a lower risk of contracting tumors than the general population [17], with an inverse comorbidity between MS and cancer. The authors also stressed that the identification of inverse comorbidity and its underlying mechanisms could provide important new insights into the understanding of MS [18]. No significant association has been found between ALS and overall cancer occurrence [11].

These epidemiologic associations are complex and challenged by confounders and exceptions [19]. Cancer treatment may also modify the relationship; some studies suggest that patients who received chemotherapy may have lower white matter organization and connectivity when compared with healthy controls [20]. Other studies suggest that chemotherapy correlates with a lower risk of AD [21]. Inverse comorbidity does not involve all types of neurodegenerative disorders and all types of cancers. Thus, the concept of inverse comorbidity, as it is discussed in this review, should always be considered in its imperfect nature rather than as a fixed rule. Nevertheless, the epidemiological data suggest a pattern of lower-than-expected combined probability of cancer and neurodegenerative diseases, which has been defined as “inverse comorbidity” [22]. The study of the mechanisms behind this pattern of inverse comorbidity could influence therapeutic interventions and provide strategies that prevent or delay both cancer and neurodegenerative diseases [19,23,24,25].

There are multiple factors that play a central role both in cancers and neurodegeneration through the same metabolic pathways that are inversely regulated and altered, as outlined in a comprehensive review published in recent years [19]. One key feature highlighted by that review was the occurrence of two patterns of association between cancer and neurodegeneration, which were summarized as “proliferation” and “apoptosis”, respectively. This implied that neurodegenerative diseases and cancers may be viewed as two sets of diseases with “too little” and “too much” apoptosis, respectively. The review also highlighted multiple factors that may underlie this difference: oxidative stress, DNA damage, inflammation, genomic instability and epigenetic alterations, mitochondrial and telomere dysfunction, metabolic dysregulation, depletion of stem cells, aberrant activation of the cell cycle, and cellular interconnections [19]. Many of these factors overlap with those that have been proposed as the pillars of aging: macromolecular damage, proteostasis, inflammation, epigenetics, metabolism, stem cells and regeneration, and adaptation to stress [26].

Another potential example of a mechanism relevant to both cancer and neurodegeneration is the non-classical, non-enzymatic binding of acetylcholinesterase (AChE) acting at an allosteric site on the nicotinic alpha-7 receptor. AChE is expressed not only in the brain but also in epithelial, endothelial, immune, and cancer cells. This form of inter-cellular communication may represent a system for triggering the entry of calcium into a wide range of cells to promote their growth. Furthermore, the AChE peptide might play a fundamental role in cell migration; therefore, through this pathway (common to neurodegeneration and carcinogenesis), there might be an interconnection between the nervous, endocrine, and immune control systems [27].

Although neurodegeneration is typical of old age, neurodevelopmental disorders might share at least some mechanisms with neurodegenerative diseases, with cognitive delay representing the counterpart of dementia. It has been estimated that 40–100% of brain tumor survivors have neurocognitive problems [28], not necessarily connected with the direct brain damage associated with the tumor and/or its treatment, and a recent review has highlighted the bidirectional correlation between cancer and neurodevelopmental disorders in pediatric age [29]. Although pediatric studies conducted on this topic are still very few, a role in the neurodevelopment deficit (especially in a fundamental period for cerebral maturation and neuronal plasticity) is played by anticancer therapies, due to their neurotoxicity [30]. Over the years, various chemotherapeutic agents used in clinical practice for the treatment of brain tumors have shown severe effects on cognitive functions, and recent studies on nanotechnology have shown that nanomaterials that could be exploited for the treatment of brain cancer are able to induce neurotoxic effects and neurodegeneration [31]. However, another important role is definitely played by inflammatory processes related to the tumor and to the considerable increase of reactive oxygen species (ROS); thus, cytokine-mediated inflammatory mechanisms might act as a trigger that initiates a cascade of events responsible for neurotoxicity [32]. In support of this, a study on children with acute lymphatic leukemia showed damage to brain structures both before and after chemotherapy, with an increase in Tau protein (suggestive of axonal damage) and in glial fibrillary acidic protein (GFAP) in patients with alterations of the apolipoprotein E gene (associated with attention deficit) and an increase in leukoencephalopathy, with impairment of the white matter [32,33].

### 1.2. The Biological Bases of the Inverse Comorbidity between Cancer and Neurodegeneration

**Figure 1:**
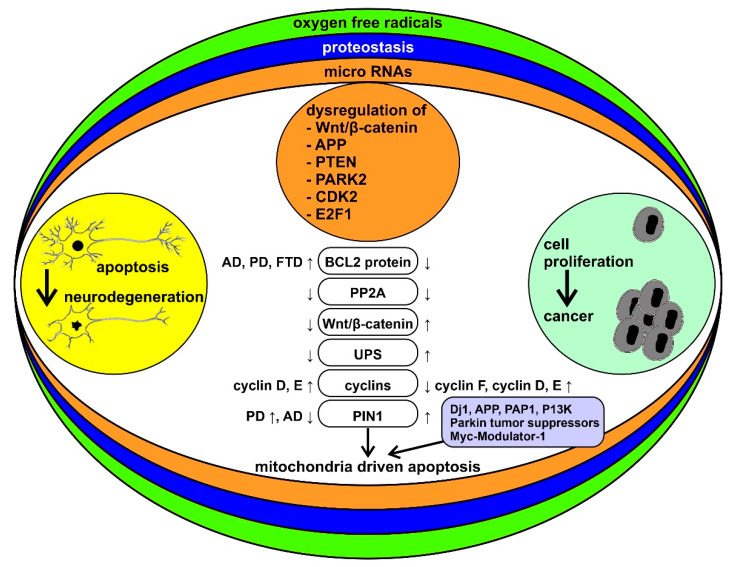
Schematic representation of the bidirectional interactions between cancer and neurodegenerative diseases and their modulatory processes. Neurodegenerative diseases and cancer are framed as two sets of disorders with “too much” or “too little” apoptosis, respectively. Reactive oxygen species (ROS), alterations in proteostasis, and microRNA (miRNAs) are among the key factors that may underlie the differences between the two sets of disorders. Some of the specific molecular mechanisms thought to be involved in this pattern of inverse comorbidity are highlighted in the middle column (see the text of the paper for abbreviations). ↓ = downregulated, ↑ = upregulated.

Inverse comorbidity between cancer and neurodegeneration may be influenced by environmental, pharmacological, and dietary factors. Genetic factors can additionally contribute to the inverse comorbidity between complex diseases [22,34,35,36]. At the base of the bidirectional interactions between cancer and neurodegenerative diseases there may be complex mechanisms that include genes, proteins, and mitochondrial function, the study of which could provide important therapeutic novelties for both diseases (Figure 1).

Mutations in PRKN (PARK2, Parkin), PARK7 (DJ-1), and PINK1 genes, which are among the genes contributing to familial cases of PD, lead to the mutation of both tumor suppressor genes, PTEN and TP53 [25]. On the other hand, PRKN and PARK5 have antiproliferative properties and are often inactivated in tumors [37]; PINK1 can also have antiproliferative functions [38]. Another mechanism potentially contributing to the inverse comorbidity is related to the brain expression of PARP1. The PARP1 protein is underexpressed in the brains of subjects with PD [39], while it is overexpressed in glioblastoma multiforme [39]. Interestingly, the PARP1 protein is also overexpressed in prostate cancer [40].

The genetic variants shared between AD and cancer are less known. Recently, a genome-wide association study (GWAS) has highlighted genes potentially involved in AD and in five different cancers (colon, breast, prostate, ovary, lung), with some shared variants modulating disease risk in a concordant way and others exerting effects in opposite directions [41]. On the other hand, the transcription factor P53 (a tumor suppressor) has multiple functions common to cancer and neurodegenerative disorders such as HD, PD, and AD [25,42], and it is crucial for cell growth control and apoptosis. Its expression is upregulated in AD, PD, and HD but downregulated in the vast majority of tumors [19,23,25].

**Figure 2:**
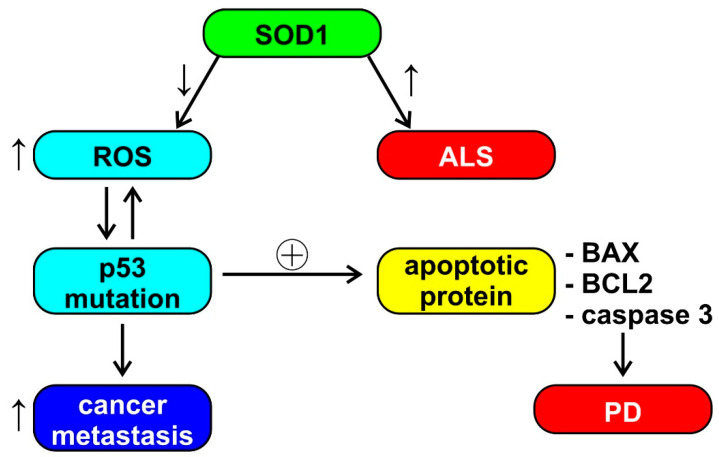
Schematic representation of the possible role of superoxide dismutase (SOD1) in amyothrophic lateral sclerosis (ALS), Parkinson’s disease (PD), and cancer metastasis. See the text of the paper for abbreviations. ↓ = decrease, ↑ = increase, ⊕ = stimulation.

Aggregation of superoxide dismutase (SOD1) causes cell death in ALS; however, SOD1 also has a role in breast cancer and the ability to increase estrogen reactive gene expression [19]. The reduced activity of SOD1 and glutathione reductase (GR) induces an increase in the production of reactive oxygen species (ROS), which leads to a conformational change of P53. The modification of P53, in turn, induces the production of ROS, thus activating oncogenic functions, such as tumor cell invasion and metastases. P53 also induces the upregulation of apoptotic proteins, such as X associated with BCL2 (BAX) and caspase 3 (observed in PD) [43]. This cascade of events, depicted also in Figure 2, seems to be an example of unclear or absent inverse comorbidity between neurodegeneration and cancer, perhaps based on the physiological and ubiquitous ROS signaling.

Interestingly, drugs used in the treatment of symptoms of neurodegenerative diseases, such as thioridazine, have been shown to have anticancer effects, while anticancer drugs, such as cyclin-dependent kinase inhibitors and mithramycin, are neuroprotective; these data reinforce the existence of a link between cancer and central nervous system (CNS) diseases and indicate that future studies will need to focus on specific molecular pathways [23].

### 1.3. The Role of Mitochondria

The BCL2 protein, involved in the mitochondrial outer membrane permeabilization, is overexpressed in CNS disorders, such as AD, PD, and frontotemporal dementia (FTD), whereas it undergoes downregulation in tumors [23]. On the other hand, cyclin D and cyclin E are overexpressed in both cancer and neurodegenerative diseases, while PP2A is downregulated in both diseases. Cyclin F is downregulated in cancer, and a mutation has been found in neurodegenerative diseases [43]. PIN1, a multifunctional gene that is hypothesized to function as a molecular timer, is overexpressed in a number of tumors and in PD but is underexpressed in AD [19,43]. Inhibition of PIN1 suppresses the growth of various tumor cells and is therefore considered as a promising therapeutic target in the oncological field [43]. PIN1, through its interactions with P53 and BCL2, can have a pro- or anti-apoptotic role depending on the cellular context. Its role in mitochondria-driven apoptosis could therefore provide a link between cancer and AD [43]. Other potentially common factors in cancer and neurodegenerative diseases include the PARK7 (DJ-1) and APP oncogenes (the first is involved in mitochondrial regulation, the second is a precursor of the β-amyloid protein), the PFDN5 (MM-1) and PRKN (Parkin) tumor suppressors (the first inhibits transcription and protein aggregation, involved in the onset of PD, cerebellar atrophy and HD, the latter plays a role in mitophagy and ubiquitination), and the PDAP1 (PAP1) and PINK1 modulators (the former is involved in splicing and the latter in mitophagy) [44].

Growing evidence suggests that age-related changes in bioenergetics at the mitochondrial level and the resulting metabolic compensation may be an important driver of both cancer and neurodegeneration and a potential target for prevention and therapy; dysfunction of mitochondria leads to disruption of DNA repair and the malfunction of metabolic pathways (such as the PI3K pathway), increasing the risk of cancer [45]. Alterations in mitochondrial DNA (mtDNA) decrease the efficiency of the respiratory chain with aging, and the prevalence of mtDNA deletions seems particularly high in neurodegenerative disorders such as PD and AD [46,47]. It has also been shown that mutations in mitochondrial DNA and alterations in mitochondrial energy metabolism can be correlated with the onset or progression of glioblastoma, following alteration of the pathways involved in apoptosis [48].

Recent data highlight an important role of the intestinal microbiome, intestinal permeability, and alterations in the functioning of mitochondria in the pathophysiology of MS: orexins, melatonin, and butyrate increase oxidative phosphorylation in mitochondria through the disinhibition of the pyruvate dehydrogenase complex, leading to an increase in acetyl-coenzyme A, a co-substrate necessary for the activation of the melatoninergic pathway of the mitochondria. Loss of mitochondrial melatonin coupled with an increase in N-acetylserotonin has implications for impaired mitochondrial function and appears to play a role in the pathophysiology of MS [49].

### 1.4. Other Factors

Telomere alterations have been recognized as a risk factor for both age-related carcinogenesis and neurodegeneration. In AD, telomere shortening has been implicated in oxidative stress and inflammation, with cognitive impairment, amyloid pathology, and hyperphosphorylation of the Tau protein. A decrease in telomere length was found in peripheral blood leukocytes in patients with AD, compared with age-matched controls, and was proposed as a potential biomarker for AD [50,51]. As introduced above, this should be considered in the framework of the imperfect inverse comorbidity between AD and cancer. Several studies have shown that telomeres shorten in the early stages of carcinogenesis and that tumor cells need to activate telomerases (which synthesize telomeres) to become immortal [52,53].

The Wnt family of secreted glycolipoprotein signaling pathway molecules takes part in the regulation of cell proliferation, polarity, and fate during the embryonic phase and in tissue homeostasis. Changes in the Wnt pathway are involved in congenital defects, cancer, and other diseases [54]. The Wnt/β-catenin signaling pathway appears to play a critical role in neural stem cell proliferation [55]. However, the dysregulation of this pathway has also been associated with cancer and neurodegenerative disorders, such as AD and PD, through an inverse correlation. In particular, the activation of Wnt signaling could be protective in neurodegenerative diseases but could promote the onset and progression of cancer [56,57]. Several molecular components of the signaling pathway have been proposed as innovative targets for cancer therapy, and very recently, some have also been evaluated as potential therapeutic targets for PD [57].

Protein homeostasis (or proteostasis) indicates the maintenance of the correct concentration of proteins of regular conformation and subcellular compartmentalization. The loss of protein homeostasis is another very important process in neurodegeneration. In particular, the family of heat shock proteins, chaperones, and the ubiquitin–proteasome system (UPS), which decrease with aging, result in aggregation of synuclein in Lewy bodies, of β-amyloid, and of Tau [58,59]. Additionally, neoplastic cells show a loss of protein homeostasis but often in the opposite direction: in cancer cells there is an overexpression of UPS and heat shock proteins [60,61]. Global hypomethylation and hypohydroxymethylation, alterations of histone proteins, and high expression of some non-coding RNAs were found in AD [62,63]. The same mechanisms are also implicated in carcinogenesis, and epigenetic therapy has already been suggested as a potential method to correct the expression levels of dysregulated genes in neurodegenerative disorders and tumors [19].

The miR-34 and miR-122 miRNAs have been used in the treatment of certain types of cancer and hepatitis, with promising results [64]. On the other hand, some miRNAs (miRNA-9, miRNA-34a, miRNA-125b, miRNA-146a, and miRNA-155) may be involved in the physiopathology of AD, and some act on the Wnt/β-catenin pathway [65], which is involved in both neurodegenerative diseases and tumors. A recent paper suggested a potential anti-β-amyloid protective effect of miRNA-15b and a biological link between miRNA-125b and neurotoxic pathways, hypothesizing that these miRNAs may play a role as biomarkers of AD physiopathology with therapeutic potential [66]. The amyloid precursor protein (APP) is connected to both AD and malignant growth. APP is concentrated in neuronal synapses and is the major component of amyloid plaques associated with AD. APP increases the proliferation and migration of epithelial cells (although the mechanism has not been fully defined) and is overexpressed in various tumors (oral cavity, esophagus, pancreatic, neuroendocrine, thyroid, and colorectal cancers) [67]. These results suggest the potential role of APP in cancer pathogenesis and reinforce our concept of imperfect inverse comorbidity between AD and cancer. Aberrant expression of miRNAs could also be involved in both neurodegeneration and tumor pathologies through the downregulation of PTEN, involved in PD. Many genes involved in both types of pathologies, including PARK2, CDK2, and E2F1, are potential targets of multiple miRNAs; however, further studies are needed to better understand their roles [67]. Taken together, these data suggest that miRNAs may be the basis for common therapeutic approaches to both cancer and neurodegenerative diseases [67,68].

## 2. Cancer, Narcolepsy, and Other Neurodegenerative Diseases

### 2.1. Narcolepsy and Cancer

Narcolepsy type 1 (NT1) is a rare neurological disease that reflects a selective loss or dysfunction of the orexin (also known as hypocretin) neurons of the lateral hypothalamus. NT1 is typically characterized by excessive daytime sleepiness and cataplexy, accompanied by sleep-wake symptoms, such as hallucinations, sleep paralysis, and sleep disturbances. Its etiopathogenesis is still under study, and a likely autoimmune genesis has recently been convincingly proposed [69]. Furthermore, a meta-analysis has highlighted an increase in serum/plasma levels of some cytokines (IL-6 and TNF-α) in patients with narcolepsy, further supporting the involvement of inflammatory mechanisms in its pathophysiology [70]. However, it should be considered that while inflammation may be involved during the disease onset period as part of an autoimmune challenge, its significance in later disease stages is under debate.

In recent years, an increased cancer risk has been reported, albeit not invariably [71], in both adults [72] and children and adolescents [73] with narcolepsy. In particular, in a study conducted on 2833 patients with narcolepsy with an observation period of 10 years, a connection with cancer risk was reported, especially for head-neck and gastrointestinal cancers and, to a lesser extent, for hematological and genitourinary cancers. The authors hypothesized genetic and immunological factors underlying the interconnection between narcolepsy and cancer [74]. These data are of interest because they suggest a link between narcolepsy and cancers in organs and tissues in which there is evidence of functional responses to orexins (brain, gastrointestinal, and genitourinary tract) [75,76,77]. Orexin might, therefore, play a role in some types of tumors as well as in NT1. However, studies on this topic are few, also due to the rarity of narcolepsy, even though they were conducted on a large registry series. On the other hand, registry data may not allow the discrimination between NT1 with orexin deficiency and narcolepsy type 2 (NT2) without orexin deficiency [73]. One study based on retrospective medical claims data found an increased comorbidity with neoplasms in both subjects with NT1 and subjects with NT2, compared with controls [72]. Thus, the epidemiological link between orexin deficiency in NT1 and cancers is still unclear and warrants further research, which may pave the way to a better understanding of these pathological processes and to new therapeutic perspectives.

### 2.2. Narcolepsy and Neurodegenerative Diseases

NT1 may coexist with AD or PD [78] or with MS [79]. The prevalence of AD in patients with NT1 was similar to that in control subjects based on postmortem brain pathology in 12 patients [80]. While these data indicate that NT1 does not confer absolute protection from neurodegenerative diseases, they are too limited to conclude for or against an association. In other studies, attention was focused on cerebrospinal fluid (CSF) neurodegeneration markers, revealing a decrease in CSF β-amyloid_1-42_ (the amyloid beta peptide with greatest propensity to aggregation) [81] in patients with narcolepsy (both NT1 and NT2) close to disease onset, which progressively recovered with time. On the other hand, patients with long disease duration of narcolepsy had higher P-Tau CSF levels than patients with short disease duration or control subjects [82]. Lower CSF levels of β-amyloid_1-42_ [83,84] and P-Tau [84] in patients with NT1 than in control subjects were reported by other studies, although not invariably [85]. Reduced brain β-amyloid burden was also detected with positron emission tomography in elderly patients with NT1, suggesting a reduced risk of progression to AD; furthermore, the authors hypothesized a protective role of orexin antagonists against neurodegeneration [86]. This suggestion is supported by preclinical evidence that in mice, the amount of β-amyloid in the brain interstitial fluid increased during orexin infusion and decreased with infusion of an orexin receptor antagonist, which also decreased β-amyloid plaque formation in amyloid precursor protein transgenic mice [87].

## 3. Orexin, Cancer, and Neurodegenerative Diseases

Although many critical pieces of evidence are still missing, the results summarized in the previous paragraphs raise the hypothesis that orexins mediate, to some extent, the links between cancer and narcolepsy and other neurodegenerative diseases. In particular, orexin deficiency is a key pathophysiological feature of patients with NT1 and might explain a greater occurrence of some types of tumors and reduced markers of neurodegenerative diseases in these patients.

To better understand this aspect, it is necessary to focus attention on the potential mechanisms of inverse comorbidity that were discussed previously, which include genes, proteins, and mitochondrial function.

### 3.1. The Orexin System

Orexin A and B (OXA and OXB) [88], also named hypocretin 1 and 2 [89], are peptides expressed by hypothalamic neurons that are most active during active wakefulness and have sparse activity during rapid-eye-movement (REM) sleep [90].

**Figure 3:**
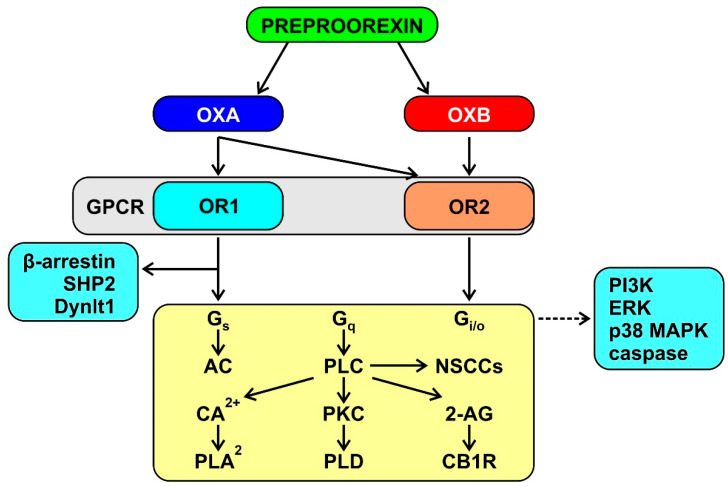
Schematic representation of the signaling potential of orexin receptors, as demonstrated by experiments on recombinant cell lines. Continuous arrows indicate demonstrated or hypothesized causal links between messengers and/or enzymes. The broken arrow indicates an undetermined causal pathway. GPCR: G-protein coupled receptors. AC: adenylate cyclase. PLC, PLA_2_, PLD: phospholipase C, A2, and D, respectively. NSCCs: non-selective cation channels. 2-AC: 2-acyl-glycerol. CB1R: cannabinoid type 1 receptor. See the text of the paper for the other abbreviations.

The orexins bind two G-protein-coupled receptors named OR1, which is selective for OXA, and OR2, which is non-selective for OXA and OXB [88]. OR1 and OR2 are widely expressed in the CNS [76], in line with the widespread projections of the orexin neurons [91]. The available evidence indicates that the orexin signaling is multifaceted and complex, with similar mechanisms elicited by OR1 and OR2 (Figure 3) [92,93]. Interpretation of this evidence is complicated by the fact that data were mostly obtained in recombinant cell lines that overexpress orexin receptors, with limited direct relevance to orexin physiology and pathophysiology. Nevertheless, the relevance of these expression systems is very considerable by implication because they reflect the intrinsic signaling capabilities of orexin receptors [94]. These capabilities underlie the in vivo modulation by the CNS orexin system of multiple physiological functions, including wake-sleep behavior, energy homeostasis, and autonomic cardiovascular control [95,96,97,98].

OR1 and OR2 are also expressed outside the CNS, including in the gastrointestinal tract, adipose tissue, male reproductive tissue [76], as well as in the heart [99] and bone marrow [77], where they may be relevant to the pathophysiology of heart failure [99] and atherosclerosis [77]. As peripheral orexin synthesis is debated [75], these receptors may bind orexins that spill over to the systemic circulation after their release in the CNS by hypothalamic neurons [77]. In this respect, it should be noted that while OXA is relatively protected from inactivating peptidases by its chemical structure, OXB is not [88] and is rapidly metabolized in blood [100] and even in CSF [101]. Any peripheral effect of circulating orexins may thus be mediated largely if not solely by OXA. However, even the blood–brain barrier permeability to OXA is still debated. OXA was reported to rapidly enter the mouse brain by simple diffusion [100], but another study reported negligible (< 1%) brain penetration of OXA in mice and rats [102].

### 3.2. Orexin and Mitochondrial Function

There seems to be no studies in the literature on the functioning of mitochondria in narcolepsy. However, a recent in vivo study reported that OXA might reduce mitochondrial biogenesis, increase mitophagy, and damage mitochondrial structure in AD. These authors also envisaged inhibition of OXA as a potential approach to the treatment of AD [103]. Similar data were reported by a study on mouse models, which showed that OXA aggravated mitochondrial deterioration, the accumulation of β-amyloid, and cognitive deficits [104]. This might imply that OXA deficit, as in NT1, would preserve the integrity and functionality of the mitochondria, reducing the risk of progression to AD. These data might also explain why NT1 and MS seem to be only rarely associated, contrary to what one would expect, since both conditions may represent autoimmune diseases [69,70,79,105]. Lack of OXA in NT1 could exert a protective effect against mitochondrial damage, preserving them from the neurodegeneration mechanisms that occur in MS [6].

Emerging studies highlight that a mitochondrial dysfunction can enhance inflammatory and immune processes, also depending on environmental factors [106].

### 3.3. Microbiota

There is promising but initial evidence that the local immune response and systemic inflammation might play a role both in tumor progression and in the survival of cancer patients [107], in neurodegeneration associated with diseases such as AD, PD, ALS, and FTD [108], as well as in NT1, a pathology with a likely autoimmune pathogenesis [69,70]. In this respect, it is also worth mentioning alterations in the structure of the intestinal microbial community that may be linked to inflammation in narcolepsy, although the data on this topic are still limited. Further broader and longitudinal studies are necessary to replicate and clarify the relationship between the gut microbiota, immunity dysregulation, and narcolepsy [109].

It seems that the intestinal microbiota plays a key role in the interaction between the brain and the gastrointestinal tract [110], due to the ability of probiotic agents to alter cytokine levels and influence brain function. The intestinal microbiota has recently been linked to the pathogenesis of neurodegenerative diseases such as PD [111] and AD [112,113], in support of a close interconnection between inflammatory, immunological, neurodegenerative, and carcinogenic processes. Recent studies suggest that the modulation of the intestinal microbiome is able to influence the immune response and numerous forms of cancer therapy by reducing their toxicity [114].

### 3.4. Genetic Factors

In a study on 426 patients with NT1, a significant association was found with increased copy number variation in the PARK2 region [115]. However, the study was conducted only on the Japanese population, and there are no data on other ethnicities. It was previously mentioned that PARK2 has antiproliferative properties and is often dysregulated due to somatic mutations in tumors [37]. A variable expression of this gene in different narcoleptic patients could therefore contribute to explanations of the increased prevalence of neoplasms in narcolepsy [74]. These findings suggest that further studies are needed on the possible genetic variations common to cancers and narcolepsy.

Mitochondria are one of the targets of miRNAs [116]; thus, alterations in miRNAs might modify mitochondrial function. Alterations in the expression of some miRNAs may be involved in the pathophysiology of narcolepsy [117]. A study revealed significant differences in the plasma levels of four miRNAs in patients with NT1 compared with healthy controls: levels of miR-30c, let-7f, and miR-26a were higher, whereas the level of miR-130a was lower in NT1. However, these differences were not specific for NT1 but also occurred in patients with NT2 or idiopathic hypersomnia. The levels of miR-26a, miR-30c, and let-7f are relatively high in the brain. miR-30c is also highly expressed in the heart, thyroid gland, blood cells, skeletal muscle, kidney, and lung. miR-26a is also expressed in the heart, thyroid, skeletal muscle, colon, and reproductive system. let-7f is also expressed in the heart, thyroid, blood cells, skeletal muscle, and reproductive system. miR-130a is expressed in the heart, lung, kidney, jejunum, colon, thyroid gland, and the reproductive system. The authors raised the hypothesis that these miRNA play a pathophysiological role and speculated that let-7f might influence T cell proliferation [118]. An alteration of miRNAs in patients with narcolepsy might be correlated with metabolic dysfunctions and carcinogenesis, especially in the gastrointestinal and genitourinary tracts, where increased tumor occurrence has been reported in these patients [74]. Very recent and interesting data indicate that miRNA specifically located in brain orexin neurons are also involved in the regulation of their viability [119].

### 3.5. Orexin in Cancer

In recent years, a series of studies have been conducted on the role of orexins not only in the field of sleep disorders but also in oncology, discovering that modulation of the orexin signaling may also unexpectedly play a therapeutic role in the treatment of some types of cancer [120,121]. One study demonstrated that OXA stimulates neovascularization [122]. Angiogenesis is the formation of new capillaries from preexisting blood vessels, a critical step in physiologic and pathologic events such as embryonic development, wound healing, chronic inflammation, and tumor growth [123]. Recent studies on the relationship between the brain and the gastrointestinal tract have suggested that OXA may play an immunomodulatory role, reducing the production of pro-inflammatory cytokines (tumor necrosis factor α, interleukin-6, and chemotactic protein of monocytes-1). Therefore, it has been proposed that the modulation of the orexinergic system may be useful in the treatment of hyperalgesia and fatigue due to chemotherapy, as well as in inflammatory bowel diseases [124].

Early work revealed a pro-apoptotic effect of OR1 signaling in colon cancer and neuroblastoma cell lines [125]. These results were later confirmed on cell lines from human colon cancer and liver metastases, both in vitro and in vivo, after xenograft in nude mice [126]. Most interestingly, OXA also promotes robust apoptosis in cells that are resistant to 5-fluorouracil, the most widely used chemotherapy in colon cancer, and reverses the development of established tumors when administered seven days after cell inoculation [125]. OXA might promote tumor apoptosis in vivo by directly activating caspase-3. These findings seem to suggest OR1 agonists for future research on colon cancer therapy [125]. More recent data on human colon cancer cell lines indicate that OXA induces autophagy [127] and that dual-agonist occupancy of OR1 and cholecystokinin A receptor heterodimers decreases migration [128]. Orexin-dependent apoptosis might be mediated by two immunoreceptor tyrosine-based inhibitory motifs in both OR1 and OR2, involvement of the phosphotyrosine phosphatase SHP2, and induction of mitochondrial apoptosis [120].

The evidence in favor of a therapeutic role of orexins on cancers other than colon cancer is more limited and partly contrasting [129]. OXA was found to suppress the growth of rat glioma cells [130]. Conversely, OXA was found to inhibit gastric cancer cell apoptosis via OR1 [131] and to enhance proliferation by upregulating the protein expression of OR1 [132], which is opposite to what was reported for colon cancer cells. Data are also contrasting for pancreatic ductal cancer and prostate carcinoma. One study reported that OR1 signaling promotes cell proliferation in pancreatic ductal cancer cells [133], whereas another study indicated that both agonism (OXA) and antagonism (almorexant) of OR1 exert an antitumoral proapoptotic effect on pancreatic ductal cancer in vitro and in vivo [134]. On the other hand, OR1 was found to be overexpressed and to mediate apoptosis in advanced prostate cancer with a neuroendocrine differentiation [135]. Accordingly, OXA administration to a human androgen-dependent prostate carcinoma cell line was later found to upregulate OR1 expression, resulting in a decrease of cell survival [136]. The same year, however, lack of expression of orexin receptors genes was reported in human normal and prostate cancer cell lines [137].

### 3.6. Orexin in Neurodegenerative Diseases

Neurodegenerative disorders may be associated with decreases in orexin neuron number and orexin system activity [97]. The orexin neuron number was found decreased by 40–72% [138,139] in the brains of patients with AD, whereas CSF OXA levels were found reduced only by 14% [138], not significantly changed [140] or even increased [141] in these patients. This discrepancy suggests either that orexin neuron loss is inconstant or that the residual orexin neurons are overactive.

PD and dementia with Lewy bodies (DLB) are neurodegenerative disorders characterized by Lewy bodies and neurites formed by alpha-synuclein deposits, whereas multiple system atrophy (MSA) is characterized by neuroglial alpha-synuclein cytoplasmic inclusions [142]. The number of orexin neurons was found dramatically reduced by 62–75% in the brain of subjects with PD, DLB, and MSA [143,144,145]). However, similar to what has been reported for subjects with AD, reductions in orexin neuron number in subjects with PD, DLB, or MSA were found generally insufficient to entail significant decreases in CSF levels of orexin [146,147,148].

OXA levels in the CSF are not significantly decreased in subjects with MS [149], unless MS entails hypothalamic lesions [150]. Nervous system inflammation is central to MS, albeit possibly secondary to neurodegeneration [5], and is also involved in the pathophysiology of the tau- and synucleinopathy neurodegenerative disorders [151,152]. Interestingly, there is evidence that OXA may decrease inflammation at neural and systemic levels. In mouse models, OXA acts on the CNS to modulate inflammation and increase survival in septic shock [153] and to alleviate inflammation after intracerebral hemorrhage [154] and in experimental immune encephalomyelitis [155]. OXA may also act on intestinal OR1 to prevent lipopolysaccharide-induced neuroinflammation at the level of the intestinal barrier [156] and to decrease inflammation in ulcerative colitis [129]. Moreover, OXA acts on OR1 bone marrow pre-neutrophils to tune down myelopoiesis, restraining the nighttime increase in circulating inflammatory monocytes and neutrophils and limiting atherosclerosis burden [77]. Thus, OXA has an immunoregulatory and neuroprotective action, inhibiting apoptosis and reducing inflammation. Moreover, OXA seems to have an action on microglia, with promising implications not only for tumors and inflammatory diseases but also for neurodegenerative diseases, although data on these are still scarce [157]. The orexin receptor antagonist suvorexant might be useful for the prevention and treatment of AD because of its neuroprotective effect, with reduction of β-amyloid plaques and improvement of synaptic plasticity [158]. At least in part, this effect may result from enhanced sleep-related brain glymphatic clearance of metabolic by-products, such as amyloid-β [159,160]. On the other hand, studies on mouse models show that orexins can ameliorate parkinsonian motor deficits by increasing the spontaneous activation of pallidal neurons [161].

## 4. Conclusions

**Figure 4:**
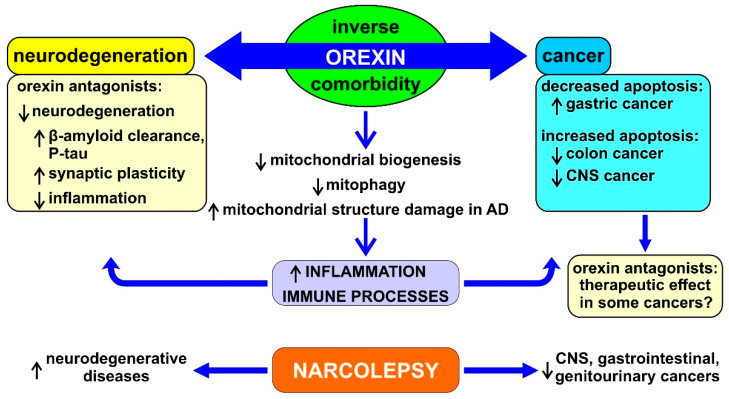
Schematic representation of the possible mechanisms underlying the role of orexin and its influences on neurodegeneration and cancer. See the text of the paper for abbreviations. ↓ = decrease, ↑ = increase.

The studies on the potential modulation of the orexin system in cancer and in neurodegenerative diseases are still pioneering and further human data are needed, although they have already shown promising results. In consideration of the concept of inverse comorbidity and in order to understand more effective therapeutic options in the future for both neurodegenerative diseases and cancer, it is important to focus on the common metabolic pathways involved in these processes. Figure 4 summarizes the possible mechanistic role of orexin in neurodegeneration and cancer.

The findings reported in this paper point to a theoretical framework in which the cell fate is determined by the (un)balance between factors favoring apoptotic or proliferative processes in reciprocal opposite directions. This ideal, although not perfect, framework has been called inverse comorbidity and indicates a lower-than-expected probability that a disease will occur in people who have another disease. A convincing amount of data have been published in recent years supporting the existence of a general inverse comorbidity between neurodegenerative diseases—such as AD, PD, MS, and others—and several types of cancer, with some exceptions. On the other hand, there are few published studies about narcolepsy in this context. Studies aimed at evaluating the role of proteins, miRNAs, and mitochondria in this area could shed more light on the etiopathogenesis of this disease, provide more answers on the biological basis of the interconnection with related pathologies, suggest new possible therapeutic perspectives, and provide new data on risk factors for the disorder.

Inverse comorbidity has its biological bases in a probably very complex mechanism in which more general and ubiquitous processes, such as ROS, miRNAs, mitochondrial function, etc., as well as more specific factors, such as orexins, play a combined role with different weights in order to favor neurodegeneration or cancer, alternatively and in mutual (quasi)exclusion.

The findings summarized in this narrative review on orexin start from epidemiological data, then include support by laboratory findings, animal models, and human clinical observational and interventional investigations. Taken together, these different lines of evidence have a relevant possible translational value, which might lead to the arrangement of novel therapeutic approaches to both neurodegenerative disease and cancer by modulating orexin pathways. This perspective warrants an important research effort on this topic in the near future.

## Data Availability

No new data were created or analyzed in this study. Data sharing is not applicable to this article.
